# Adding salt to the mix: The m^6^A reader ECT8 is a stress sensor, expediting mRNA decay in Arabidopsis

**DOI:** 10.1093/plcell/koae156

**Published:** 2024-05-24

**Authors:** Regina Mencia

**Affiliations:** Assistant Features Editor, The Plant Cell, American Society of Plant Biologists; Facultad de Bioquímica y Ciencias Biológicas, Instituto de Agrobiotecnología del Litoral (CONICET-UNL), Cátedra de Biología Celular y Molecular, Universidad Nacional del Litoral, 3000 Santa Fe, Argentina

How do plants respond to their environment? How is it possible for a plant to thrive in diverse environmental conditions? What are the molecular signals and mechanisms that regulate these processes? These questions occupy the minds of numerous scientists seeking answers.

When plants encounter stress, a molecular revolution is triggered at all levels, from gene transcription to protein regulation. At the epigenetic level, RNA modifications emerge as a crucial point. Chemical modifications of RNA, particularly *N*^6^-methyladenosine (m^6^A), have been shown to mediate several processes, including chromatin maintenance, RNA metabolism, translation efficiency, and various other biological events (Hu et al. 2022).

Upon stress, particularly salt stress, there is a dynamic change in m^6^A levels (Hu et al. 2021). However, the mechanism governing this phenomenon and the extent of its meaning remain elusive. The epigenetic responses implicating m^6^A may involve m^6^A writers, erasers, and readers (Hu et al. 2022). In Arabidopsis (*Arabidopsis thaliana*), there are 13 predicted m^6^A readers, among which ECT2, ECT3, and ECT4 have been associated with leaf morphogenesis and abscisic acid responses (Arribas-Hernández et al. 2018; Song et al. 2023). In this issue, Zhihe Cai, Qian Tang, Peizhe Song, and their colleagues (Cai et al. 2024) elucidate the role of the Arabidopsis m^6^A reader ECT8 as an abiotic stress sensor (see Fig.).

**Figure.**
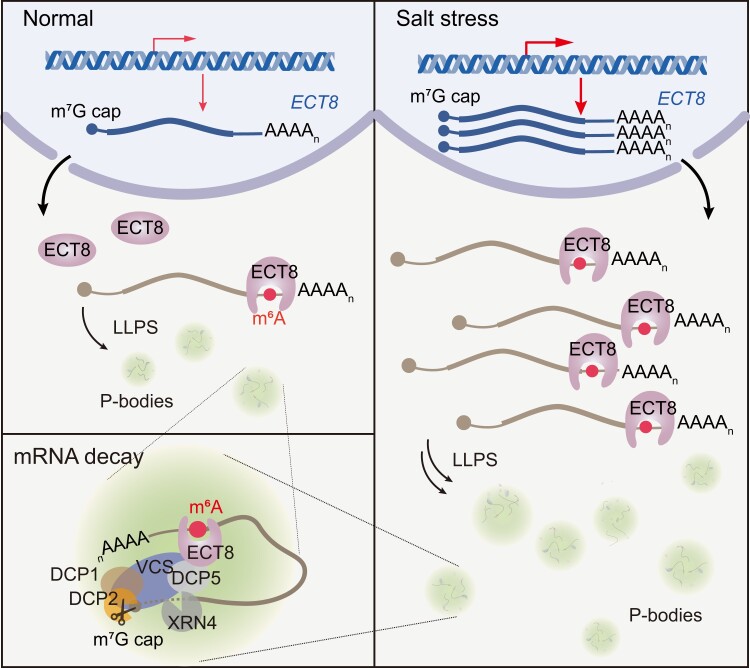
ECT8 acts as an m^6^A reader, hastening the degradation of its bound m^6^A-modified mRNA by directly interacting with DCP5 in P-bodies. The increased presence of ECT8 protein upon environmental stress intensifies the degradation of m^6^A-modified mRNA. Reprinted from Cai et al. (2024), Figure 7.

When the authors exposed plants to salt stress, they observed a transcriptional induction of *ECT8*, accompanied by protein accumulation, contrasting with other analyzed m^6^A writers or erasers and indicating a specific involvement in this stress response. Notably, the positive regulatory role of ECT8 in salt response was assessed by the heightened sensitivity to stress in knockout mutants. Particularly, in FA–cross-linking and immunoprecipitation data, ECT8 was found to bind mostly to the 3'UTR of genes involved in pathways related to RNA metabolism, gene expression regulation, and significantly enriched in genes involved in response to salt stress.

Comparing the protein structure of ECT8 with mammalian homologs revealed the possible significance of 3 conserved tryptophan residues. The authors confirmed their importance for ECT8 function experimentally by creating a mutant version with point mutations in 2 of the 3 tryptophans. EMSA assays, along with in vitro and in vivo RNA immunoprecipitation followed by liquid chromatography-MS, demonstrated the capacity of ECT8 to bind m^6^A RNA and its dependence on the presence of the tryptophan residues. Even more, the mutant version of ECT8 incapable of binding m^6^A displayed increased sensitivity to salt stress.

These findings suggest a positive role for ECT8 in salt response, but how does ECT8 perform this role? To answer this question, the authors examined the transcripts bound by ECT8. They found that upon stress, the amount of m^6^A in these transcripts does not change, but ECT8's binding ability to their m^6^A region increases. Additionally, the absence of ECT8 results in longer transcript half-lives. Considering the protein structure with the presence of a disordered Prion-like domain, it was hypothesized that ECT8 could engage in liquid-liquid phase separation and localize into processing bodies (P-bodies), which are cytoplasmic protein complexes involved in mRNA degradation. Indeed, it was confirmed that ECT8 localizes to P-bodies and directly interacts with the P-body component Decapping 5 (DCP5).

Taking all the evidence into account, the authors unveiled the role of ECT8 as a salt stress sensor, facilitating mRNA decay and contributing to the understanding of epitranscriptomic gene regulation (see Figure). Further investigation is needed to decipher the missing pieces of the puzzle, such as what controls the induction of *ECT8* and which primary signal triggers the mechanism.

## Data Availability

There is no data associated or generated from this article.

## Dive Curated Terms

The following phenotypic, genotypic, and functional terms are of significance to the work described in this paper:

ECT8 Gramene: AT1G79270
ECT8 Araport: AT1G79270

ECT2 Gramene: AT3G13460
ECT2 Araport: AT3G13460

ect3 Gramene: AT5G61020
ect3 Araport: AT5G61020

ect4 Gramene: AT1G55500
ect4 Araport: AT1G55500
